# Amyloid Precursor Protein Mediates Neuronal Protection from Rotenone Toxicity

**DOI:** 10.1007/s12035-018-1460-7

**Published:** 2019-01-05

**Authors:** Kathryn Cimdins, Hayley S. Waugh, Vicki Chrysostomou, M. Isabel G. Lopez Sanchez, Vanessa A. Johannsen, Mark J. Cook, Jonathan G. Crowston, Andrew F. Hill, James A. Duce, Ashley I. Bush, Ian A. Trounce

**Affiliations:** 1 Centre for Eye Research Australia, Royal Victorian Eye and Ear Hospital, Melbourne, Victoria Australia; 2 Ophthalmology, Department of Surgery, The University of Melbourne, 75 Commercial Road, Melbourne, Victoria 3004 Australia; 3 Department of Oncology, University of Oxford, Oxford, UK; 4 Department of Medicine, St. Vincent’s Hospital, The University of Melbourne, 75 Commercial Road, Melbourne, Victoria 3004 Australia; 5 Centre for Clinical Neuroscience and Neurological Research, St. Vincent’s Hospital, Melbourne, Victoria Australia; 6 Department of Biochemistry and Genetics, La Trobe Institute for Molecular Science, La Trobe University, Melbourne, Victoria Australia; 7 The ALBORADA Drug Discovery Institute, Cambridge Biomedical Campus, University of Cambridge, Cambridge, UK; 8 The Florey Institute of Neuroscience and Mental Health, The University of Melbourne, Melbourne, Victoria Australia

**Keywords:** Mitochondria, Amyloid precursor protein, Neuroprotection, Rotenone, Complex I, Retina

## Abstract

Mitochondrial complex I dysfunction is the most common respiratory chain defect in human disorders and a hotspot for neurodegenerative diseases. Amyloid precursor protein (APP) and its non-amyloidogenic processing products, in particular soluble APP α (sAPPα), have been shown to provide neuroprotection in models of neuronal injury; however, APP-mediated protection from acute mitochondrial injury has not been previously reported. Here, we use the plant-derived pesticide rotenone, a potent complex I-specific mitochondrial inhibitor, to discover neuroprotective effects of APP and sAPPα in vitro, in neuronal cell lines over-expressing APP, and in vivo, in a retinal neuronal rotenone toxicity mouse model. Our results show that APP over-expression is protective against rotenone toxicity in neurons via sAPPα through an autocrine/paracrine mechanism that involves the Pi3K/Akt pro-survival pathway. APP^−/−^ mice exhibit greater susceptibility to retinal rotenone toxicity, while intravitreal delivery of sAPPα reduces inner retinal neuronal death in wild-type mice following rotenone challenge. We also show a significant decrease in human retinal expression of APP with age. These findings provide insights into the therapeutic potential of non-amyloidogenic processing of APP in complex I-related neurodegeneration.

**Electronic supplementary material:**

The online version of this article (10.1007/s12035-018-1460-7) contains supplementary material, which is available to authorized users.

## Introduction

Mitochondrial complex I (NADH/ubiquinone oxidoreductase) is the main entry point of electrons into the respiratory chain from carbohydrate metabolism, via the oxidation of NADH. Complex I is the largest of the five oxidative phosphorylation (OXPHOS) enzymes, comprising 45 protein subunits including 7 encoded in the mitochondrial DNA [1, 2]. Within mitochondrial disorders, complex I defects account for a disproportionate majority of disease, which range in clinical severity from Leber hereditary optic neuropathy with specific retinal ganglion cell loss, to severe multisystem progressive and lethal encephalopathies [2–4]. Partial systemic complex I defects have been demonstrated in Parkinson’s disease [5], glaucoma [6, 7], and in Leber hereditary optic neuropathy [8]. The plant-derived pesticide rotenone is a potent complex I-specific inhibitor that is widely used to model Parkinson’s disease and optic neuropathies both in vitro and in vivo [9–11].

The primary clinical presentations in Parkinson’s disease are motor symptoms such as bradykinesia and resting tremor, but the disease also routinely involves non-motor symptoms such as visual and olfactory deficits. Recent work has evidenced retinal thickness as an accessible biomarker given that thinning of the retinal layer is an early event in Parkinson’s disease [12] and continued thinning corresponds both with the progression of disease symptoms [13] and nigral dopaminergic loss [14]. It is generally accepted that the causal origin of Parkinson’s disease is unknown in most cases. A series of molecular mechanisms, including mitochondrial dysfunction, oxidative stress, excitotoxic damage, and loss of protective proteins, are hypothesized as early, contributing, interacting, and/or causal events [15].

The amyloid precursor protein (APP) is the parent protein of a complex family of derivative peptides that exert various neuroprotective activities [16, 17]. APP is predominantly processed from its membrane-bound holoform via two pathways. The favored non-amyloidogenic pathway produces a large N-terminal ectodomain, soluble APP α (sAPPα), which is secreted into the extracellular medium [18], and a C-terminal fragment, which undergoes further processing to generate a peptide called p3 [19]. Alternatively, amyloidogenic APP processing results in the release of a shorter ectodomain called soluble APP β into the extracellular medium, and a 99-amino acid C-terminal fragment in the membrane [20]. Further cleavage of this C-terminal fragment generates the amyloid β (Aβ) peptide found in cerebral plaques of neuropathological diseases such as Alzheimer’s disease [21, 22].

Non-amyloidogenic APP processing has been shown to provide neuroprotection in models of neuronal injury, including traumatic brain injury and ischemia-reperfusion injury [23–25]. APP-mediated protection from acute mitochondrial injury has not been previously described. Here, we use the plant-derived pesticide rotenone, a potent complex I-specific inhibitor, and Parkinson’s disease mimetic, to discover neuroprotective effects of APP and sAPPα in vitro and in vivo and provide mechanistic insights into the role of APP in counteracting mitochondrial neurotoxic stress.

## Materials and Methods

### Cell Culture and Plasmid Transfection

Human neuroblastoma SH-SY5Y, mouse neuroblast neuro2-a (N2a), or Chinese hamster ovary (CHO) cells were grown in RPMI 1640 medium (Cat. #11875, Thermo Fisher Scientific) and supplemented with 10% fetal bovine serum (FBS) in a humidified incubator at 37 °C and 5% CO_2_. Cells were transfected with a pIRESpuro2 empty vector control (Clontech Laboratories Inc., Takara-Bio Inc., Japan) or a vector containing human wild-type full-length APP_695_ isoform (hereafter referred to as “control” or “APP” respectively) as described previously [26]. Cells were transferred to glucose-free RPMI 1640 medium (Thermo Fisher Scientific, Cat. #11879) supplemented with 7.5% FBS and 5.6 mM glucose (euglycemic medium) 24 h prior to rotenone treatment, because hyperglycemia can selectively reduce physiological α-secretase cleavage of APP [27].

### Ethics

All procedures conformed to the requirements of the Royal Victorian Eye & Ear Hospital Animal Research and Ethics Committee. Wild-type and APP^−/−^ mice on a SV129 background [28] originated from a breeding colony at the Melbourne Brain Centre, Melbourne VIC, Australia. Mice were housed in a temperature (22 ± 1 °C) and light (12 h light, 12 h dark) controlled environment where food and water were available ad libitum. Male and female mice were used equally.

### Human Eye Collection and Processing

Work involving human eyes was carried out in accordance of the Declaration of Helsinki. All experimental procedures were approved by the Royal Victorian Eye and Ear Hospital Human Research Ethics Committee (Project 08/859H) and written informed consent was obtained from all next of kin or participants. Human eyes were donated for research purposes through the Lions Eye Donation Service (Melbourne, Australia). Information was received in relation to the donor’s medical history including cause of death, time of death, past illnesses and any relevant eye history if known.

### Experimental Treatments and Co-culture

Rotenone (Sigma-Aldrich) was dissolved in ethanol and potassium cyanide (KCN; Sigma-Aldrich) was dissolved in ultrapure MilliQ water. Wortmannin (Sigma-Aldrich; 20 nM; wmn) was added to cells 1 h before rotenone treatment. Co-culture of SH-SY5Y control and APP cells was achieved using a ThinCert apparatus (Greiner Bio-One, USA, 1 μm membrane).

Briefly, control SH-SY5Y cells were seeded in 24-well culture plates (main well) and control or APP-over-expressing SH-SY5Y cells were plated into Thincert inserts (insert; Greiner Bio-One, USA) for 40 h prior to rotenone exposure. Co-culturing occurred for 16–8 h prior to rotenone treatment to allow secreted APP fragments to move freely between culture populations by diffusion while preventing direct interaction of both cell types.

### In Vivo Rotenone Toxicity Model

Mice were anesthetized by intraperitoneal injection of ketamine (60 mg/kg) and xylazine (10 mg/kg). After making a guide track through the conjunctiva and sclera at the superior temporal hemisphere using a 30-gauge needle, a hand-pulled glass micropipette was inserted into the mid-vitreal cavity. Rotenone (1 μl; 10 mM; Sigma-Aldrich) or vehicle (DMSO) was injected into the vitreal chamber at a rate of 100 nl/s using an Ultra Micro Pump (World Precision Instruments, Inc. Sarasota, USA). Patency was confirmed following needle removal. For sAPPα co-treatment experiments, a second intravitreal injection of recombinant human sAPPα (6 ng/μl, Sigma-Aldrich) or vehicle (phosphate-buffered saline; PBS) was performed 30 min after injection of rotenone.

### Cell Viability and Caspase 3 Assay

Cell viability and proliferation were measured by the trypan blue exclusion assay. Cells seeded at equal densities were scraped and collected by centrifugation (1000*g*, 5 min), suspended in PBS, and a 10-μl aliquot mixed with an equal volume of 0.4% trypan blue solution (Cat # 1525006, Thermo Fisher Scientific) and counted using a hemocytometer. For caspase 3 activity analysis, cells were collected following rotenone treatment and lysed in lysis buffer (50 mM HEPES, pH 7.4, 150 mM NaCl, 1 mM EDTA, 0.2 mM EGTA, 1% Triton X-100, 1 mM phenylmethylsulfonyl fluoride, 10 μg/ml aprotinin, 5 μM Na_3_VO_4_), passed twice through a 21 G needle, incubated on ice for 10 min, and the supernatant collected by centrifugation (10, 000*g*, 10 min). A 50-μl aliquot of this lysate was used to measure caspase-3 activity using the EnzChek Caspase-3 Assay kit (Molecular Probes, USA) according to manufacturer’s instructions.

### Reactive Oxygen Species Generation

ROS were measured using 2,7-dichlorodihydrofluorescein (DCFH)—to detect hydrogen peroxide—and dihydroethidine (DHE)—to detect superoxide, with a Polarstar fluorescence microplate reader (POLARstar OPTIMA, BMG laboratories, Australia) by measuring fluorescence with excitation and emission wavelengths of 480 ± 10 and 570 ± 10 nm, respectively. Briefly, cells were plated at 5 × 10^4^ in 96-well plates in euglycemic medium for 24 h prior to treatment for up to 24 h, media was removed, and cells incubated in the dark with DCFH or DHE for 30 min at 37 °C. Cells were then washed twice with PBS and suspended in PBS for fluorescence analysis. A total of 100 nM phorbol 12-myristate 12-acetate dissolved in DMSO was used as a positive control for superoxide detection, while 1 mM H_2_O_2_ was used as a positive control for hydrogen peroxide detection.

### ATP Synthesis

ATP quantification was carried out using the ATP Determination Kit Assay (Cat. # A22066, Thermo Fisher Scientific) according to manufacturer’s instructions, with a Polarstar fluorescence microplate reader (POLARstar OPTIMA, BMG laboratories, Australia).

### Protein Extraction to Determine Phosphorylation State

Harvested cells were collected by centrifugation (1000*g*, 3 min, 4 °C) and suspended in lysis buffer containing 50 mM Tris-HCl pH 7.4, 150 mM NaCl, 1 mM EDTA, 1% Tergitol, 0.1% SDS in milliQ H2O, 2 mM activated sodium orthovanadate (Na_3_VO_4_), 1 mM (PMSF), and aprotinin 10 μg/ml. Samples were sonicated on ice (3 pulses at power setting 1.5, 0.04 V, Milsonix homogenizer, Milsonix Inc., USA) and snap-frozen at − 80 °C for 2 h. Lysed cells in the supernatant were collected by centrifugation (1000*g*, 30 s, 4 °C).

### Immunoblotting

Harvested cells, retina, or vitreous were suspended in 70–100-μl lysis buffer, sonicated on ice (as described above), and incubated on ice for 30 min, followed by centrifugation (18,000*g*, 20 min, 4 °C) to collect the supernatant containing protein. Immunoblotting was performed as described before [29] and protein expression was detected using primary antibodies: anti-APP 22C11 (# MAB348, Millipore, 1:5000), anti-sAPPα 2B3 (#11088, IBL, 1:2000), anti-Akt (#9272, Cell Signaling, 1:5000), anti-phospho-Akt Ser473 (#9271, Cell Signaling, 1:2000), anti-phospho-Akt Thr308 (#9275, Cell Signaling, 1:2000), anti-phospho-ERK1/2 (Cell Signaling, 1:2000), anti-phospho-JNK (Cell Signaling, 1:1000), anti-phospho-p38 (Cell Signaling, 1:1000), anti-actin antibody (Sigma-Aldrich, 1:5000). Primary antibodies were detected using sheep anti-mouse or anti-rabbit horseradish peroxidase-conjugated secondary antibody (Amersham GE Healthcare, Cat # NA931V or #NA934) and visualized by electrochemiluminescence detection reagent on film (Amersham GE Healthcare, Cat # RPN2106). Protein band intensities were measured using ImageJ software (https://imagej.nih.gov/ij/), and band intensity determined in the linear range was normalized to band intensity of actin.

### Collection and Processing of Mouse Retinal Tissue

Eyes were enucleated and immersion-fixed in 4% paraformaldehyde for 3 h, followed by overnight cryoprotection in 15% sucrose. Eyes were embedded in optical cutting temperature medium and 12 μm sections were cut through the papillary-optic nerve axis.

### Mouse Retina Imaging and TUNEL Assay

Cryosections were immunolabeled with an antibody that recognizes the N-terminal of APP (22C11, 1:100, Merck Millipore) according to published protocols [30]. All sections were nuclear-counterstained with Hoechst (1:10,000). The thicknesses of cellular and synaptic retinal layers were measured on digital images of Hoechst-stained cryosections as described [31]. To quantify retinal ganglion cells, sections cut through the optic nerve head and ora serrate were scanned from superior to inferior edge and the numbers of Hoechst-labeled nuclei in the ganglion cell layer were counted. Retinal cryosections were labeled using the TUNEL assay according to published protocols [32]. Labeled sections were scanned from superior to inferior edge in 1-mm increments and the number of TUNEL-positive nuclei were recorded. The frequency of TUNEL-positive profiles/mm of retina was averaged from at least two sections per animal.

### Statistical Analysis

Results are presented as the mean ± SD or ± SEM (indicated in the legend). Statistical analysis was performed with Prism 5.01 software (GraphPad Software Inc.) using Student’s *t* test, one-way ANOVA with Bonferroni correction for multiple comparisons or linear regression. Normal distribution of experimental measurements was determined by the Shapiro-Wilk test. A *P* value < 0.05 was considered as significant.

## Results

### APP Protects Against Rotenone Toxicity in Neuronal Cells

**Fig. 1:**
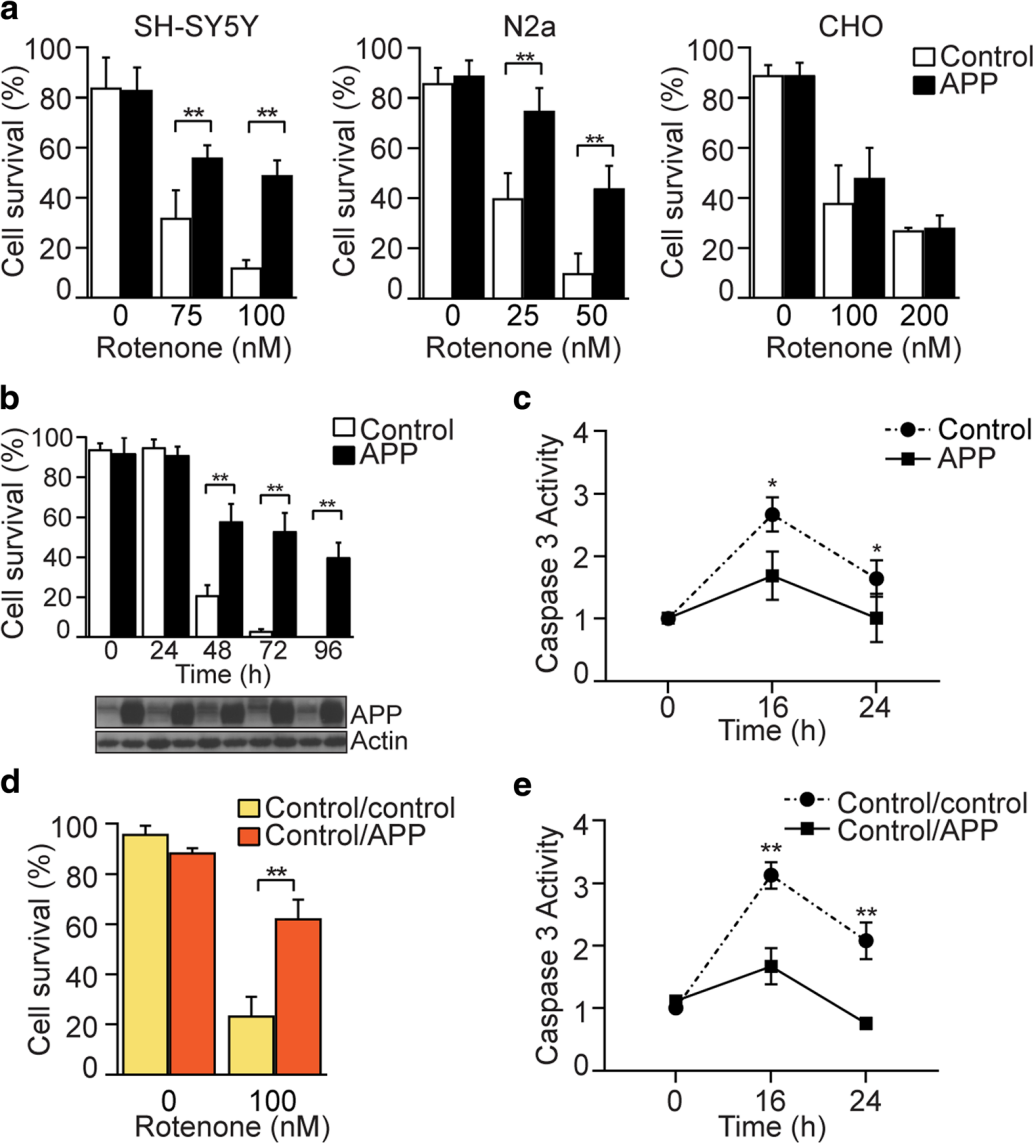
APP protects against rotenone toxicity in neuronal cells. **a** Human neuroblastoma SH-SY5Y, mouse neuroblastoma N2a or non-neuronal CHO cells transfected with APP (APP) or empty vector control (control) were exposed to increasing concentrations of rotenone for 48 h and cell survival was determined by the trypan blue exclusion assay. SH-SY5Y and N2a cells transfected with APP had a significantly higher survival compared to control. **b** Cell survival in transfected SH-SY5Y cells exposed to 100 nM rotenone was assessed up to 96 h to show sustained protection in SH-SY5Y cells expressing APP. Representative immunoblot showing increased APP levels in SH-SY5Y cells exposed to rotenone at each timepoint is included below the graph. Actin was used as a loading control. **c** Caspase 3 activity is significantly higher in control SH-SY5Y cells 16 and 24 h post-exposure to 100 nM rotenone compared to SH-SY5Y cells expressing APP. **d** Co-culture of control SH-SY5Y with SH-SY5Y cells expressing APP cells increases resistance to rotenone toxicity. Control SH-SY5Y cells were co-cultured with either SH-SY5Y APP cells (control/APP) or control (control/control) for 16 h before incubation with vehicle or rotenone (100 nM) for 48 h and cell survival was determined by the trypan blue exclusion assay. **e** Caspase 3 activity is significantly higher in control/control cells compared with control/APP cells 16 and 24 h post-exposure to 100 nM rotenone. Data show mean ± SD, *n* ≥ 4, Student’s *t* test, * P < 0.05, ** P < 0.01.

We investigated the effect of APP over-expression in the loss of neurons upon exposure to the mitochondrial neurotoxin and Parkinson’s disease mimetic rotenone [33, 34]. Human neuroblastoma SH-SY5Y, mouse neuroblastoma N2a, or non-neuronal CHO cells were transfected with an empty vector (control) or a vector containing full-length, wild-type APP_695_ (APP). APP protein levels were measured by immunoblotting in SHSY-5Y, N2a, and CHO cells to demonstrate higher APP expression relative to control cells (Supplementary Fig. 1A). Transfected cell lines were exposed to increasing concentrations of rotenone for 48 h and cell survival was determined by the trypan blue exclusion assay (Fig. 1a). APP over-expression correlated with reduced cell loss in both SH-SY5Y and N2a cells compared to control cells, while no changes in cell survival were detected in CHO cells over-expressing APP. This protective effect was confirmed in transfected SH-SY5Y cells exposed to 100 nM rotenone up to 96 h, showing sustained protection in neuronal cells expressing higher levels of APP (Fig. 1b). APP protein levels were consistently higher in SH-SY5Y cells transfected with APP upon exposure to increasing concentrations of rotenone (Supplementary Fig. 1B). This was consistent with significantly higher activation of caspase 3 in control cells compared with APP over-expressing cells at 16 and 24 h following rotenone challenge (Fig. 1c).

It is well established that sAPPα, resulting from the non-amyloidogenic cleavage of APP and released into the extracellular medium, is neuroprotective and neurotrophic [16]. To determine the paracrine neuroprotective potential of APP against rotenone-induced cell loss, control SH-SY5Y cells were co-cultured with control cells (control/control) or with APP cells (control/APP) for 16 h prior to rotenone exposure, in a transwell culture system that allowed secreted APP fragments to move freely between culture populations but prevented direct interaction of the different cell types (Supplementary Fig. 1C). Control/control or control/APP co-cultures were exposed to rotenone (100 nM) for 48 h and cell survival was determined by the trypan blue exclusion assay (Fig. 1d). sAPPα levels were increased in conditioned media from control/APP co-cultures as measured by immunoblotting (Supplementary Fig. 1D). Cells continually exposed to sAPPα-enriched media through co-culture were significantly protected against rotenone-induced cell loss compared to control/control cells (Fig. 1d). This was further explored by measuring caspase 3 activity, which was significantly higher in control/control co-cultures compared with control/APP co-cultures, indicating that an activation of apoptotic pathways in control/control cells is absent in control/APP cells (Fig. 1e).

Next, we investigated the specificity of APP-mediated protection from mitochondrial toxicity, particularly OXPHOS complex IV inhibition, which has also been associated with age-related neurodegeneration [35]. Transfected cells were exposed to increasing concentrations of the complex IV inhibitor potassium cyanide (KCN) for 48 h and cell survival was assessed by the trypan blue exclusion assay (Supplementary Fig. 2). Unlike the complex I inhibition experiments, APP over-expression did not protect neuronal or non-neuronal cells from complex IV inhibition-induced toxicity (Supplementary Fig. 2A) or caspase 3 activation (Supplementary Fig. 2B). Furthermore, cellular ATP levels decreased at similar levels between control and APP transfected cells upon exposure to potassium cyanide (Supplementary Fig. 2C). Together, these results indicate that APP confers protection to neuronal cells from complex I toxicity induced by exposure to rotenone through an autocrine (i.e., intracellular APP over-expression) or paracrine (i.e., derived from secreted APP in co-culture) mechanism. This protection appears to be neuron-selective and specific to complex I.

### APP Prevents Rotenone-Induced Changes in Mitochondrial ROS and ATP Production

**Fig. 2:**
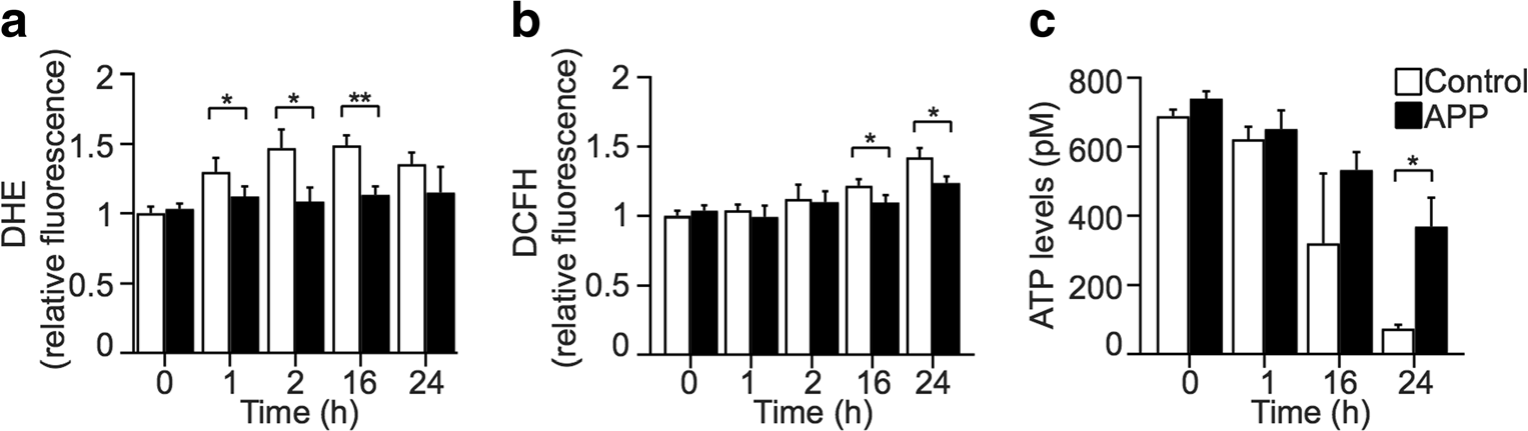
APP alters rotenone-induced changes in mitochondrial ROS and ATP production. **a**, **b** Levels of cellular superoxide (**a**; DHE fluorescence) and hydrogen peroxide (**b**; DCFH fluorescence) were assessed in SH-SY5Y control and APP cells treated with 100 nM rotenone for up to 24 h. **c** Over-expression of APP in suppression of rotenone-induced levels of superoxide and hydrogen peroxide in APP over-expressing SH-SY5Y cells has delayed effects on ATP cellular levels. Data show mean ± SD, *n* ≥ 3, Student’s *t* test, *P < 0.05, **P < 0.01

Rotenone inhibits complex I of the mitochondrial electron transport chain and induces apoptosis via reduction of ATP synthesis and increased ROS production [36]. Therefore, to identify the mechanism of APP-mediated neuroprotection from rotenone toxicity in mitochondria, ATP and ROS levels were examined in control or APP SH-SY5Y cells incubated with 100 nM rotenone for up to 24 h (Fig. 2). Over-expression of APP suppressed superoxide levels significantly between 1 and 16 h (Fig. 2a) and peroxide levels at 16 and 24 h following rotenone challenge (Fig. 2b). Basal ATP levels were unchanged by the over-expression of APP relative to control cells up to 16 h; however, at 24 h post-rotenone exposure, ATP levels were preserved at significantly higher levels in cells over-expressing APP compared with control (Fig. 2c). These results reflect a delayed effect of APP on mitochondrial metabolism that coincide with caspase 3 activation and loss of plasma membrane integrity in control cells (Fig. 1).

### Pi3K/Akt Activation Is Crucial to APP-Mediated Neuronal Protection from Rotenone Toxicity

**Fig. 3:**
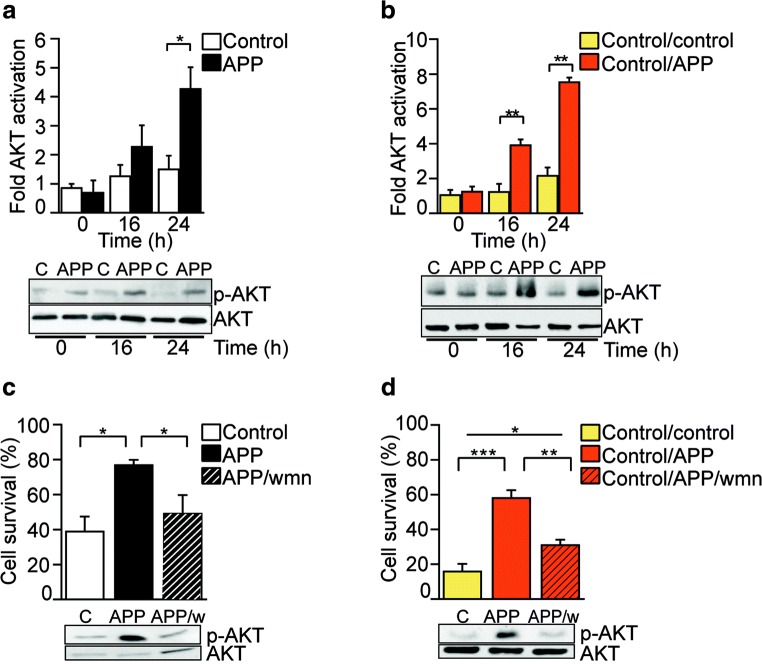
Akt activation is crucial to APP-mediated neuronal protection from rotenone toxicity. **a**, **b** In response to rotenone (100 nM) exposure, Akt was activated in SH-SY5Y cells over-expressing APP (**a**) and in SH-SY5Y control/APP co-cultures (**b**). **c** Cell survival was assessed in SH-SY5Y control or APP cells treated with rotenone (75 nM; 48 h) in the absence or presence of the Akt inhibitor wortmannin (w, wmn, 20 nM). **d** Cell survival was determined in SH-SY5Y control/control or control/APP co-cultures exposed to rotenone (75 nM; 48 h) in the absence or presence of wortmannin (w, wmn, 20 nM). Representative blots and densitometric analyses show the ratio of activated (phosphorylated, p-AKT) protein levels relative to unphosphorylated protein levels. Data show mean ± SEM, *n* ≥ 3, Student’s *t* test (**a**, **b**) or one-way ANOVA (**c**, **d**). *P < 0.05, **P < 0.01, ***P < 0.001

Exposure of neuronal cultures to sAPPα has been shown to activate the Pi3K/Akt pro-survival pathway and thereby prevent apoptosis following trophic factor deprivation [37, 38], Aβ toxicity [39], or proteasome inhibition [40]. To further investigate the neuroprotective mechanism in our model of rotenone-induced mitochondrial toxicity, Akt phosphorylation response was measured by immunoblotting in control or APP SH-SY5Y cells (Fig. 3a), and in control/control or control/APP co-cultures (Fig. 3b) upon exposure to rotenone for up to 24 h. APP over-expression alone or exposure to sAPPα-enriched media progressively induced significant and sustained Akt activation, reaching up to a 3-fold increase relative to controls by 24 h (Fig. 3a, b). Next, we assessed the functional relevance of Akt activation in the survival associated with APP over-expression or exposure to sAPPα-enriched media, by blocking Akt activation using the Pi3K inhibitor wortmannin (wmn; Fig. 3c, d). We observed a consistent and significant reversal in cell survival after 24-h exposure to rotenone in both APP over-expressing cells (Fig. 3c) and control/APP co-cultures upon treatment (Fig. 3d) in the presence of wortmannin, demonstrating that Akt activation is crucial for neuronal protection in our experimental setting.

Others have shown that rotenone-induced apoptosis requires activation of MAPK signaling pathways such as JNK and p38 [38, 41]. Therefore, we investigated whether protection from rotenone toxicity in cells over-expressing APP could also result from suppression of pro-apoptotic targets upstream of Akt. Over-expression of APP did not significantly alter the rotenone-induced acute activation of p38 (Supplementary Fig. 3A), JNK (Supplementary Fig. 3B), or c-Jun (Supplementary Fig. 3C). Furthermore, over-expression of APP did not alter rotenone-induced activation of the pro-survival ERK signaling pathway (Supplementary Fig. 3D). Together, these results suggest that the mechanism of APP-mediated neuroprotection against rotenone is through the Pi3K/Akt pro-survival pathway and independent of MAPK or ERK pathways.

### APP Protects Against Rotenone-Toxicity in the Mouse Retina

**Fig. 4:**
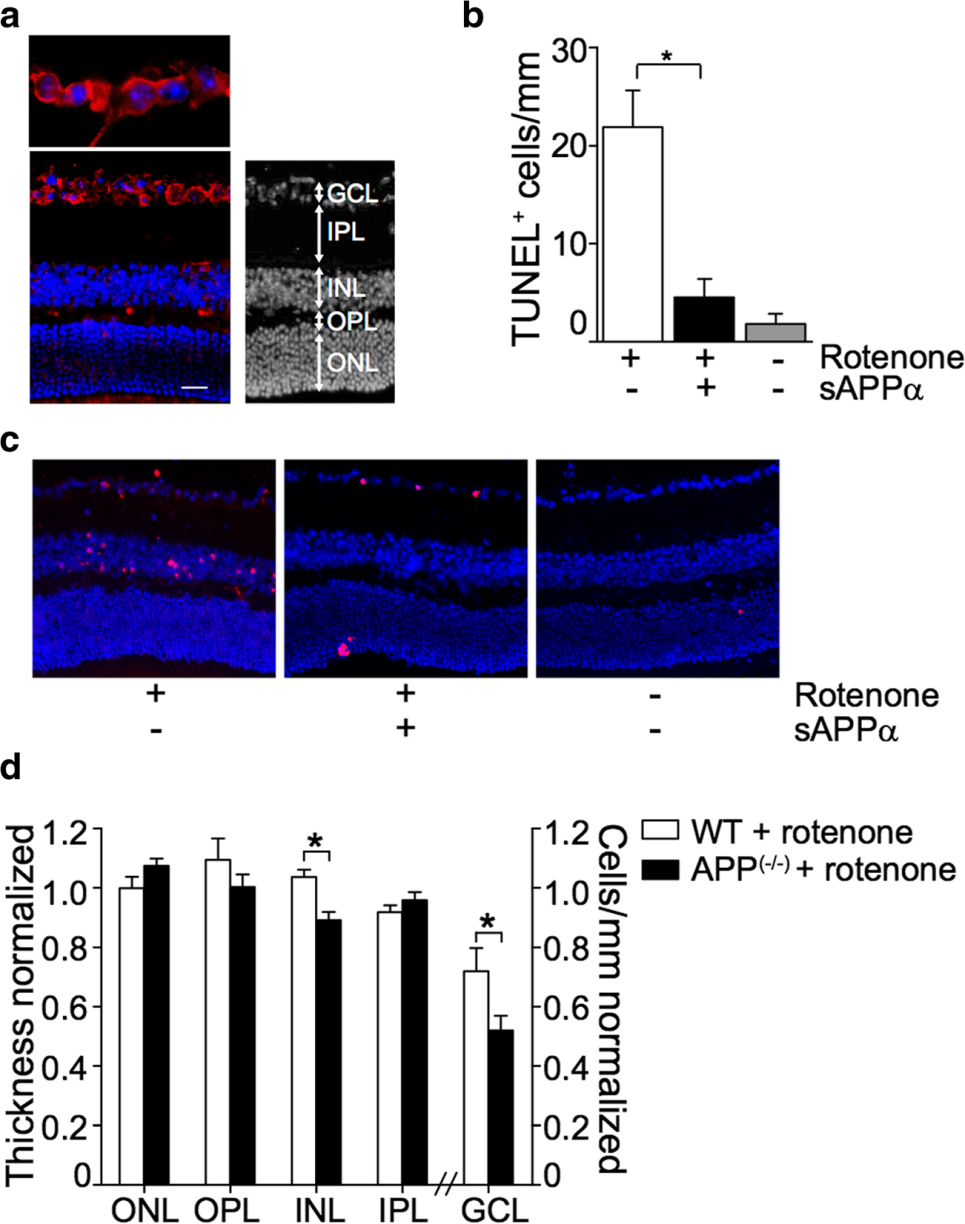
APP protects against rotenone-toxicity in the mouse retina. **a** Expression of APP in the mouse retina was assessed by immunohistochemistry of cross-sections using an antibody that detects all APP isoforms and APP fragments (22C11). Representative images show localization of APP (red) in the retinal ganglion cell layer. Scale bar = 20 μm. **b** Cell death in the mouse retina was quantified by the frequency of TUNEL-positive nuclei in the inner retina 24 h after rotenone toxicity. Data are presented as mean ± SEM; *n* = 8; *p < 0.05 by Student’s *t* test. **c** Representative micrographs of mouse retinal cross-sections show TUNEL-positive cells (red). Scale bar = 20 μm. **d** Survival of retinal neurons 24 h after rotenone toxicity in wild-type (WT) and APP knockout (APP^−/−^) mice was quantified by measuring the thickness of nuclear and synaptic layers and by counting the number of soma in the ganglion cell layer on nuclear-stained retinal cross-sections. Data are presented as mean ± SEM; *n* = 8; *p < 0.05 by Student’s *t* test. ONL: outer nuclear layer; OPL: outer plexiform layer; INL: inner nuclear layer; IPL: inner plexiform layer; GCL: ganglion cell layer

We next sought to determine if the neuroprotective effect of APP or sAPPα in vitro extended to an in vivo setting. APP immunoreactivity in the C57BL/6J mouse retina was most prominent in somas of the ganglion cell layer (GCL) on the inner retinal surface (Fig. 4a, in red), concurring with previous reports [42]. Intravitreal delivery of rotenone is an established model of retinal toxicity in the mouse eye that selectively kills inner retinal neurons [11]. Using this model, we assessed mouse retinas 24 h after rotenone injection by the TUNEL technique, which identifies DNA damage characteristic of apoptosis, to estimate cell death. We found a significant increase in the frequency of TUNEL-positive nuclei in the ganglion and inner nuclear layers of rotenone-treated eyes compared to those injected with vehicle (Fig. 4b, c), confirming the toxicity model and its specificity to the inner retina. To test if sAPPα was able to rescue retinal neurons against rotenone toxicity, we injected exogenous sAPPα into the mouse eye 30 min post-rotenone injury. Administration of sAPPα significantly reduced the number of TUNEL-positive profiles in the mid and inner layers of the retina (Fig. 4b, c). There was no detectable difference in the number of TUNEL-positive cells between sAPPα-treated retinas and uninjured (vehicle-injected) retinas.

If APP is critical for retinal cell survival after toxic challenge, we hypothesized that mice deficient in APP would show a greater susceptibility to rotenone-induced toxicity. To test this hypothesis, we conducted experiments with mice carrying a homozygous deletion of the APP gene (APP^−/−^ mice). These mice have no detectable levels of APP mRNA or protein but are fertile and do not show overt abnormalities up to 12 weeks of age [28]. Initial histological analysis of baseline retinal cross-sections revealed no overt structural abnormalities in APP^−/−^ mice (Supplementary Fig. 4). Since we established that rotenone treatment-induced cell loss was localized to the inner nuclear and ganglion cell layers of the retina, analysis on the response of APP^−/−^ mice to rotenone toxicity was focused specifically on these locations. On retinal cross-sections collected 24 h after rotenone treatment, we quantified soma in the ganglion cell layer and assessed the integrity of the inner nuclear layer by measuring its thickness. Compared to wild-type, APP^−/−^ mice showed a significantly greater loss of retinal ganglion cell layer (GCL) in response to rotenone injection (Fig. 4d). Furthermore, the inner nuclear layer (INL) of APP^−/−^ mice was significantly thinner than that seen in wild-type mice, indicating a greater loss of inner retinal cell bodies in response to rotenone (Fig. 4d). There was no change in the thickness of the outer nuclear layer (ONL), or plexiform layers (OPL/IPL) of the retina in either wild-type or APP^−/−^ mice with rotenone, indicating no substantial loss of photoreceptors or synaptic connections (Fig. 4d). Collectively, these findings support our in vitro findings and suggest a neuroprotective role for sAPPα in vivo.

### Age-Related Changes to APP Levels in the Human Eye

**Fig. 5:**
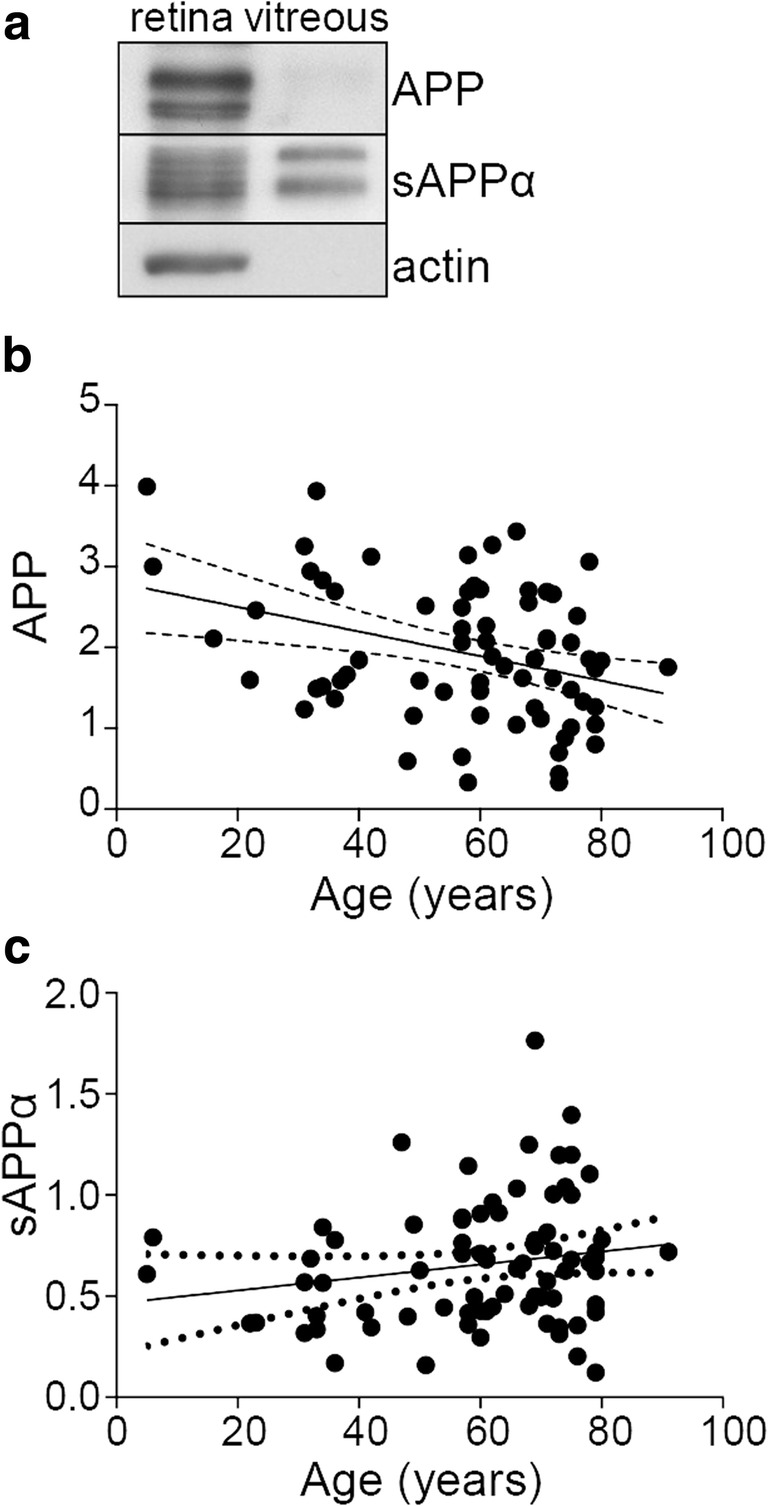
APP levels decrease in the aging human eye. **a** Representative image of APP expression in the human eye, assessed by immunoblotting using antibodies against full-length APP (anti-APP 22C11; Millipore) and sAPPα (anti-sAPPα 2B3; IBL). Both species of APP were detected in the retina while only sAPPα was detected in the vitreous. **b**, **c** Linear regression analysis of retinal APP (**b**) and retinal sAPPα (**c**) levels and age in the human eye

There is consistent and substantial evidence for a neuroprotective function of APP and sAPPα in vivo [16], which is further supported by our current data. However, due to the difficulty in obtaining clinical samples, most of the in vivo work to date has used rodent models and there is little data available on APP and sAPPα in human eye tissue. A previous study using immunoreactivity analysis in a small number of human eyes showed that APP is predominantly located in the inner retina, specifically the ganglion cell and retinal nerve fiber layers [43]. We conducted an analysis of APP and sAPPα protein levels in retinal tissue and vitreous fluid from a large cohort of human eyes collected over a 3-year period through the Lions Eye Donation Service, Melbourne, Australia (Table 1). Retinal tissue was collected from donors that ranged in age from 5 to 91 years of age (*n* = 83), while vitreous fluid was obtained from donors ranging from 22 to 91 years of age (*n* = 41). Immunoblotting was performed using antibodies that detect full-length APP (#A8717, Sigma) or sAPPα (anti-sAPPα 2B3, IBL). We detected the presence of both full-length APP and sAPPα in human retinal samples (using actin as a loading control) and confirmed only sAPPα in the vitreous samples (Fig. 5a). An absence of full length APP and actin (Fig. 5a) indicated the purity of the vitreous samples and the lack of contamination by retina or other tissue during dissection. By quantifying retinal APP and retinal sAPPα levels, a linear regression analysis was used to address the relationship between retinal expression and age in the human eyes. While there was a significant decrease in retinal expression of APP with age (Fig. 5b), no change was seen in the levels of retinal sAPPα (Fig. 5c).

**Table 1:** Characteristics of donors for aging retina and vitreous analysis

Sample type	Subset (years)	*n*	Sex (female *n*; %)	Age (average, years)
Retina	total	83	38 (45.8)	59.2 ± 18.9; range 5–91
	< 40	16	7 (43.8)	27.9 ± 10.6
	40–70	37	18 (48.6)	58.8 ± 8.2
	> 70	30	13 (43.3)	76.4 ± 4.9
Vitreous	Total	41	18 (43.9)	62.4 ± 16.5; range 22–91
	< 40	6	2 (33.3)	32 ± 5.3
	40–70	18	8 (44.4)	59.2 ± 7.4
	> 70	17	8 (47.1)	76.6 ± 6.2

## Discussion

Here, we investigated the neuroprotective effects of APP and sAPPα in vitro and in vivo, using models of neurodegeneration induced by rotenone exposure, and report a loss in retinal APP with age in humans. APP confers protection to neuronal cells from rotenone through both autocrine and paracrine mechanisms mediated via the Pi3K/Akt pro-survival pathway. The addition of sAPPα to primary neuronal cultures under trophic factor deprivation has been shown to reinstate the Pi3K/Akt and ERK pathways and provide protection from cell death [37, 38]. By contrast, the activation of Akt in APP over-expressing cells and cells exposed to sAPPα-enriched media found here was insult-driven. This correlated with the relative preservation of ATP levels as well as the repression of cellular peroxide levels and caspase 3 activity in APP over-expressing cells compared to control cells. Over-expression of APP did not affect rotenone-induced p38 or JNK pathway activation despite sAPPα-mediated JNK suppression being linked to its neuroprotective mechanism against epoxomicin [40]. It did, however, immediately and continually suppress rotenone-induced superoxide levels, which may be a result of the reported doubling of manganese superoxide dismutase levels in APP over-expressing neuroblastoma cells [44].

One reason over-expression of APP protected against rotenone-induced mitochondrial complex I dysfunction and not cyanide-induced mitochondrial complex IV dysfunction may be linked to Akt targets. The cyanide model used here was caspase 3-independent and resulted in a rapid loss of cellular ATP. By promoting the interaction of hexokinase with mitochondria and inhibiting Bax translocation to mitochondria [45], activated Akt effectively prevents mitochondrial cytochrome *c* release and subsequent caspase 3 activation. This pro-survival pathway would therefore be ineffective against the rapid cyanide-induced cell death observed here.

Using an in vivo model of acute rotenone toxicity in the mouse retina, we showed that intravitreal delivery of recombinant human sAPPα, which lacks the posttranslational modifications present in endogenously processed sAPPα, reduces retinal neuronal cell death in wild-type animals. The same toxicity model in APP^−/−^ mice resulted in a greater loss of inner retinal neurons compared to wild-type mice, suggesting that endogenous APP processing provides some protection from the complex I inhibition. Our finding of abundant levels of sAPPα in human vitreous implicates this neurotrophin in retinal homeostasis. We and others [42] show that retinal ganglion cells have the highest expression of APP in the wild-type mouse retina, which is the most likely source of sAPPα in the vitreous. Examination of retinal APP protein levels in a large cohort of human eyes illustrate a significant decrease with age, raising the question of whether a loss in APP contributes to age-related neurodegeneration.

The accumulation of Aβ is a traditional hallmark of Alzheimer’s disease but other forms of neurodegeneration, including a subset of patients with Parkinson’s disease with dementia, have a similar neuropathological observation [46]. However, as the generation of Aβ or sAPPα arise from alternative proteolytic pathways of APP it is reasonable to propose that an increase in Aβ production will result in a decrease in sAPPα levels [47]. Increased Aβ production thereby results in both a gain of Aβ toxicity and a potential loss of sAPPα neurotrophic/neuroprotective function. Lower sAPPα levels have been found in cerebro-spinal fluid of Alzheimer’s disease patients carrying the Swedish mutations in APP [48] and in post mortem brain [49], but pre-mortem studies in cerebro-spinal fluid or blood in patients with sporadic Alzheimer’s disease have not provided consistent results [50]. Few studies have investigated APP levels in Parkinson’s disease brain. However, Ayton et al. showed lower APP levels in post mortem Parkinson’s disease substantia nigra, and mice over-expressing APP are protected from MPTP-induced nigral cell loss [51]. This is consistent with our findings as both rotenone and the neurotoxin MPP^+^ (derived from MPTP) are potent mitochondrial complex I inhibitors.

## Conclusions

In summary, we show that APP over-expression or exposure to sAPPα affords protection from mitochondrial complex I inhibition by rotenone. This protection is driven by Pi3K/Akt activation. Our finding that sAPPα can protect from acute rotenone toxicity provides therapeutic insights into mitochondrial neurodegeneration. Pharmacological mimetics of this protection mechanism could be promising therapeutic candidates in diseases resulting from complex I impairment including mitochondrial optic neuropathies and Parkinson’s disease.

## Electronic supplementary material

ESM 1 (PDF 929 kb)

## Abbreviations

Akt: protein kinase B
APP: amyloid precursor protein
Aβ: amyloid beta
DCFH: 2,7-dichlorodihydrofluorescein
DHE: dihydroethidine
ERK: extracellular-signal-regulated kinase
FBS: fetal bovine serum
GCL: ganglion cell layer
INL: inner nuclear layer
IPL: inner plexiform layer
JNK: c-Jun N-terminal kinase
MAPK: mitogen-activated protein kinase
MPP^+^: 1-methyl-4-phenylpyridinium
MPTP: 1-methyl-4-phenyl-1,2,3,6-tetrahydropyridine
NADH: nicotinamide adenine dinucleotide + hydrogen
ONL: outer nuclear layer
OPL: outer plexiform layer
OXPHOS: oxidative phosphorylation
PBS: phosphate-buffered saline
Pi3K: phosphatidylinositol-3-kinase
ROS: reactive oxygen species
sAPPα: soluble amyloid precursor protein alpha
TUNEL: terminal deoxynucleotidyl transferase dUTP nick-end labeling
Wmn: wortmannin
