# Supplementary material for: Amyloid Precursor Protein Mediates Neuronal Protection from Rotenone Toxicity
Source: Mol Neurobiol. 2019 Jan 5;56(8):5471–82. doi: 10.1007/s12035-018-1460-7 (PMC6614131; doi:10.1007/s12035-018-1460-7)
Supplement: Supplementary file 1 — (PDF 929 kb) [file 12035_2018_1460_MOESM1_ESM.pdf]

Amyloid precursor protein mediates neuronal protection from rotenone toxicity

Kathryn Cimdins, Hayley S. Waugh, Vicki Chrysostomou, M. Isabel G. Lopez Sanchez, Vanessa A. Johannsen, Mark J. Cook, Jonathan G. Crowston, Andrew F. Hill, James A. Duce, Ashley I. Bush, Ian A. Trounce*

Journal Title: Molecular Neurobiology

Corresponding Author

Associate Professor Ian Trounce

75 Commercial Road, Melbourne, Victoria 3004, Australia. Tel.: +61 3 8532 1964; E-mail address: i.trounce@unimelb.edu.au

Supplementary Material

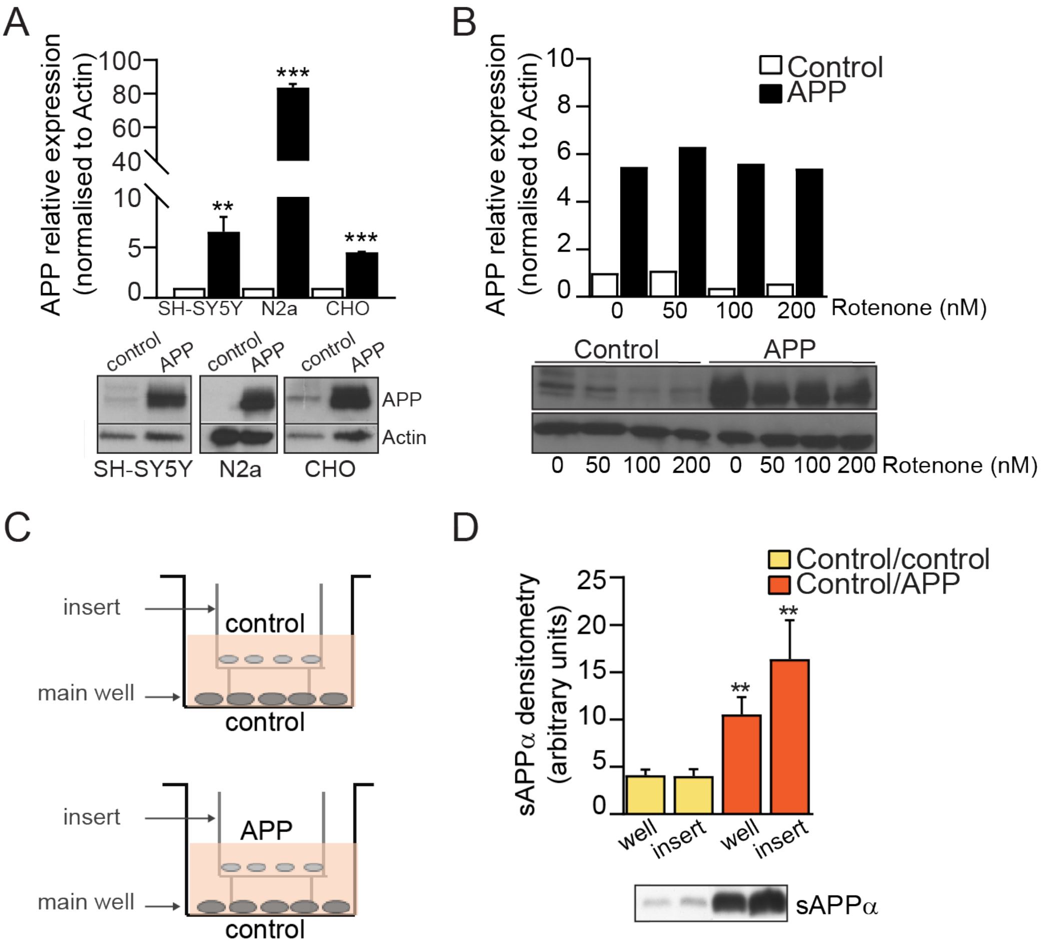

Supplementary Figure 1. APP and sAPPα levels in transfected cells

A. APP protein levels are higher in SHSY-5Y, N2a and CHO cells transfected with APP relative to cells transfected with an empty vector control. Actin was used as a loading control. **B.** APP levels are increased in SH-SY5Y APP cells exposed to increasing concentrations of rotenone for 48 h. Actin was used as a loading control. **C.** Schematic of co-culture experimental setting. Control SH-SY5Y cells were seeded in 24-well culture plates (main well) and control or APP-over-expressing SH-SY5Y cells were plated into Thincert inserts (insert) to allow secreted APP fragments to move freely between culture populations by diffusion while preventing direct interaction of both cell types.

D. sAPPα levels are higher in medium collected from the main well or insert from control/APP co-cultures. Data show mean ± SD, n ≥ 4, Student's *t* test, *** P < 0.01*, **** P < 0.001*.

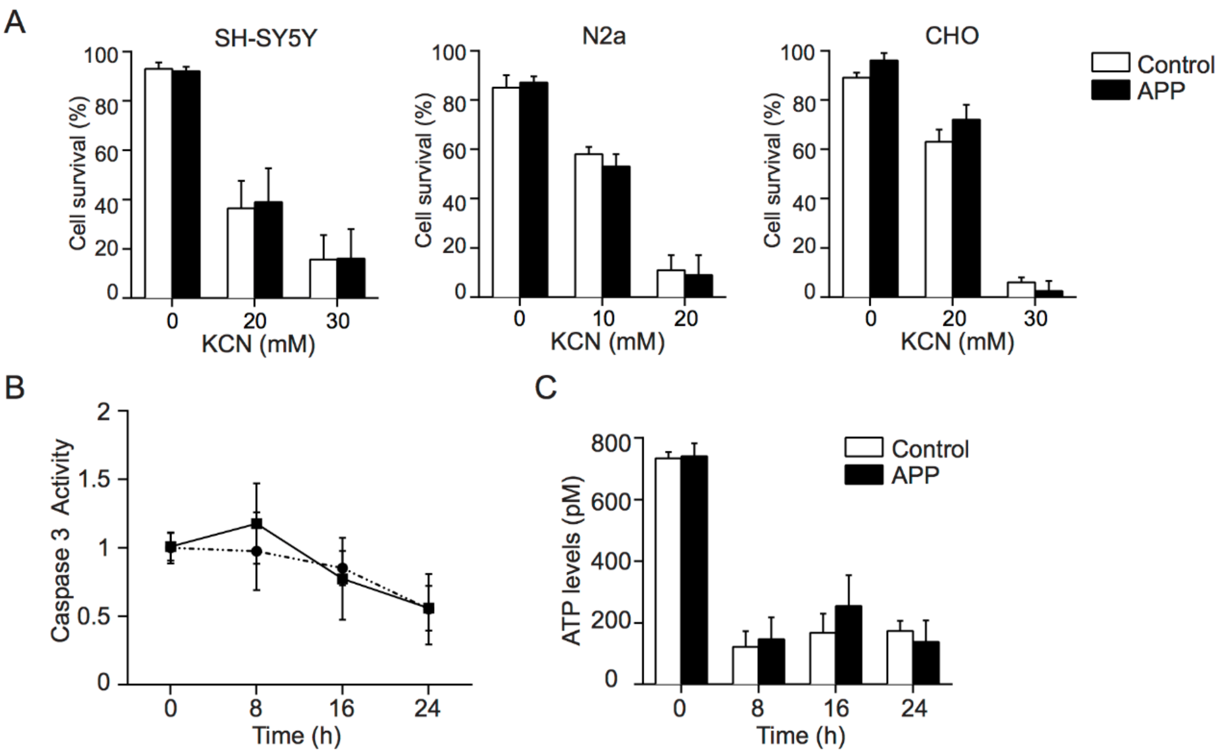

Supplementary Figure 2. APP does not protect against cyanide toxicity.

A. Human neuroblastoma SH-SY5Y, mouse neuroblastoma N2a or non-neuronal CHO cells transfected with APP (APP) or empty vector control (control) were exposed to increasing concentrations of potassium cyanide (KCN) for 48 h and cell survival was determined by the trypan blue exclusion assay. **B**. Caspase 3 activity is not altered in SH-SY5Y cells expressing APP relative to control SH-SY5Y cells following exposure to KCN. **C.** ATP cellular levels decrease at similar levels in control and APP SH-SY5Y cells upon exposure to KCN. Data show mean ± SD, n ≥ 3, Student's *t* test.

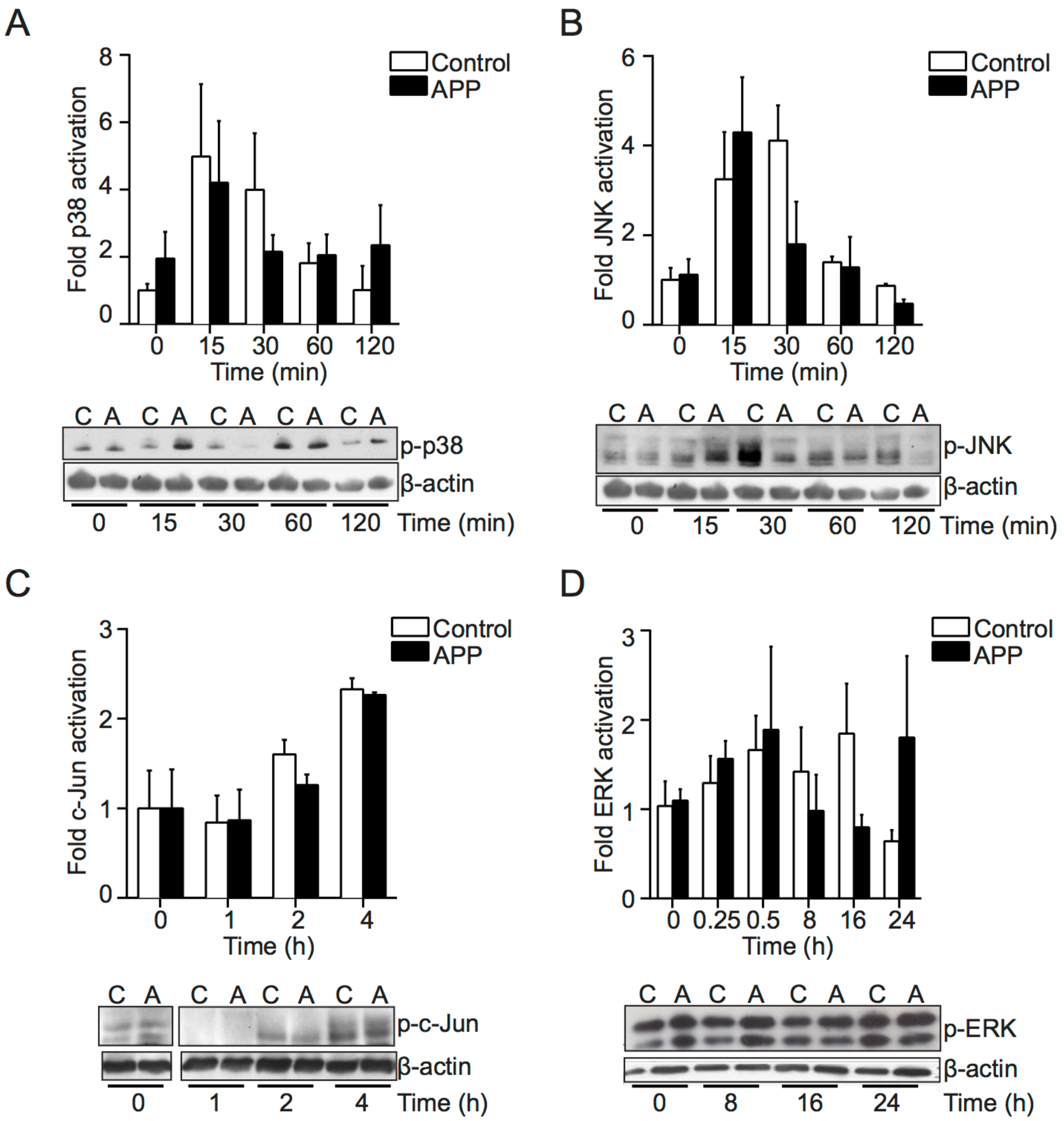

Supplementary Figure 3. MAPK signaling does not mediate APP-associated protection from rotenone toxicity.

Over-expression of APP in SH-SY5Y cells did not significantly alter the rotenone-induced activation of p38 (**A**), JNK (**B**), c-Jun (**C**) or ERK (**D**) signaling pathways. Representative blots and densitometric analyses show activated (phosphorylated, p-) protein levels compared to basal protein levels of control cells. Data show mean ± SEM, n ≥ 4, Student's *t* test.

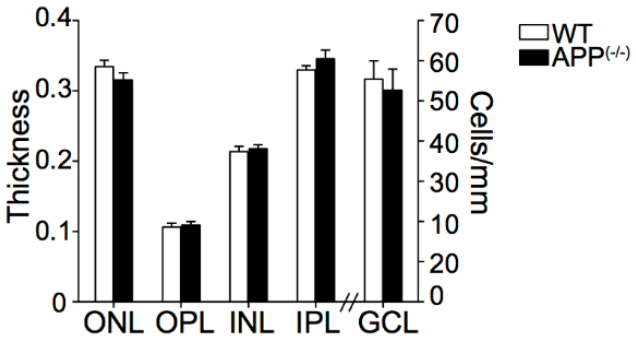

Supplementary Figure 4. Retinas from the APP$^{-/-}$ mouse do not have overt structural abnormalities.

Histological analysis of baseline retinal cross-sections revealed no overt structural abnormalities in APP$^{-/-}$ mice. ONL: outer nuclear layer; OPL: outer plexiform layer; INL: inner nuclear layer; IPL: inner plexiform layer; GCL: ganglion cell layer.
